# Oral antibiotics prior to colorectal surgery: Do they have to be combined with mechanical bowel preparation?

**DOI:** 10.1017/ice.2019.157

**Published:** 2019-08

**Authors:** Tessa Mulder, Jan A.J.W. Kluytmans

**Affiliations:** 1 Julius Center for Health Sciences and Primary Care, University Medical Center Utrecht, Utrecht University, Utrecht, The Netherlands; 2 Department of Infection Control, Amphia Hospital, Breda, The Netherlands

## Abstract

To reduce the of risk infection after colorectal surgery, oral antibiotic preparation (OAP) and mechanical bowel preparation (MBP) can be applied. Whether OAP can be used without MBP is unclear. A meta-analysis of observational studies demonstrated comparable effectiveness of OAP with and without MBP regarding SSI risk.

Surgical site infection (SSI) is a common complication after colorectal surgery. To reduce the risk of SSI, oral antibiotic preparation (OAP) and mechanical bowel preparation (MBP) can be administered before surgery. Usually, these 2 prophylaxes are combined because of their presumed synergistic effect. The combination has been shown to reduce the risk of SSI compared to no preparation, but it is unknown to what extent each of the preparations contribute to this decline. MBP was abandoned recently due to a lack of evidence for a beneficial effect compared to no preparation.^1,2^ Together with the ban of MBP, OAP was also discarded, although its efficacy without MBP was never investigated. Because SSI risk after colorectal surgery remains high, there has been a resurgence of interest in bowel preparation. A recent meta-analysis pooled all evidence from randomized controlled trials (RCTs) to determine whether no preparation, MBP, OAP, or MBP and OAP combined is the most effective in preventing postoperative complications.^3^ The combination of MBP and resulted in the lowest risk of SSI. An important limitation is that it was not possible to conclude whether OAP is effective without MBP because no RCTs have focused only on OAP. In this study, we aimed to provide insight into the effectiveness of OAP without MBP on SSI risk using data from observational studies.

## Methods

We performed a systematic review and meta-analysis of observational studies that investigated OAP prior to colorectal surgery. We searched PubMed on ‘oral antibiotic bowel preparation’ and MeSH terms ‘colorectal surgery’ and ‘surgical wound infection,’ and we included studies that investigated an OAP only strategy. Data on study design, data-analysis, and the number of SSI per preparation strategy were collected. Because we aimed to reduce confounding bias, we extracted the adjusted odds ratios (aORs) and 95% confidence intervals (CIs). We pooled the aORs for the comparisons of OAP only versus no preparation and OAP with MBP versus no preparation. A random effects model was used to account for the expected clinical heterogeneity due to known variation in OAP. When studies were performed on (a subset of) the same cohort, we only included the study with the highest precision in the meta-analysis to ensure that patients were included in the meta-analysis only once. Statistical analyses were performed with Review Manager software.

## Results

**Fig. 1.**
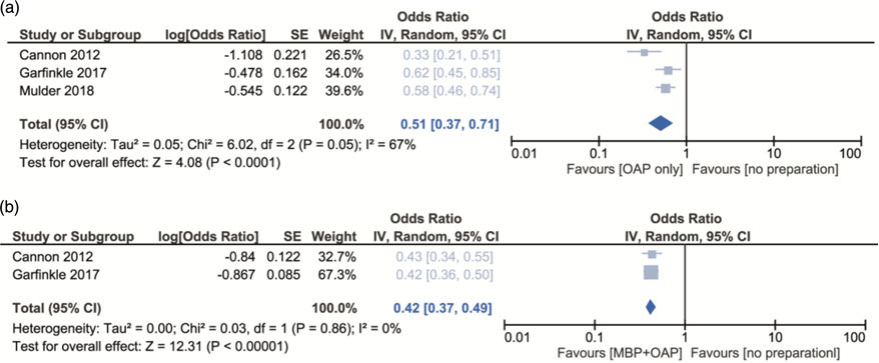
Forest plots of adjusted odds ratios for the outcome all SSI of the following associations: (A) OAP versus no preparation and (B) OAP and MBP combined versus no preparation. Because of suspected overlap in participants between several studies, only the largest of the ACS-NSQIP database studies was included to calculate the pooled effect.

We found 15 studies that reported data on OAP without MBP (Table 1).^4–18^ In almost all studies, the OAP strategy only was the least often used preparation. Because 13 studies were performed with data from the ACS-NSQIP database from 2012 through 2015, a substantial overlap in participants was suspected, and only the largest study was included in the meta-analysis. The forest plots with pooled aORs are presented in Fig. 1. Compared to no preparation, SSI risk was significantly reduced when patients received either OAP only (aOR, 0.51; 95% CI, 0.37–0.71) or OAP combined with MBP (aOR, 0.42; 95% CI, 0.37–0.49). The largest study reported no significant difference between MBP with OAP versus OAP alone (aOR, 0.78; 95% CI, 0.55–1.08).

**Table 1.** Observational Studies on Antibiotic Bowel Preparation

Author, Year and Country	Study Period	Patients	Study Design	Statistical Methods	Confounders Adjusted For	Type of SSI	Bowel Preparation Strategy No. Patients, No. SSI (%)				aOR (95% CI)		
							No Prep	MBP Only	OAP Only	MBP+OAP	OAP Only vs No Prep	OAP+MBP vs No Prep	OAP+MBP vs OAP Only
ACS-NSQIP database studies													
Scarbourough 2012, USA	2012	Elective colorectal	Retrospective cohort	Logistic regression analysis	BMI, diabetes, smoking, COPD, hypertension, chemotherapy, disseminated cancer, weight loss, albumin, surgical approach, wound class, operative time, total work relative units, low pelvic anastomosis	Incisional	N=1,092 98 (9.0)	N=2,322 174 (7.5)	N=91 4 (4.4)	N=1,494 48 (3.2)	0.41 (0.15–1.17)	0.33 (0.23–0.47)	
Althumairi 2016, USA	2012–2013	Elective colorectal	Retrospective cohort	Logistic regression analysis; Model 1: bowel prep Model 2: bowel prep + SSI Model 3: confounders + bowel prep Model 3: confounders, bowel prep + SSI	Age, sex, race, ASA classification, smoking status, diabetes, history of congestive heart failure, history of COPD, BMI, weight loss, indication for surgery, surgical approach, type of procedure, operative time.	All Used as independent variable, not as outcome	N=5,060 692 (13.7)	N=8,020 922 (11.5)	N=641 54 (8.4)	N=5,965 374 (6.3)	*No aOR for SSI provided*		
Atkinson 2015, USA	2012–2013	Elective colorectal	Retrospective cohort	Logistic regression analysis	Age, diabetes, smoking, operative time, blood transfusion, steroids, ASA classification, surgical approach, indication for surgery, wound class	All	N=5,741 786 (13.7)		N=658 64 (9.7)		0.66 (0.48–0.90)		
Moghadamyeg-haneh 2015, USA	2012–2013	Elective colorectal;	Retrospective cohort	Logistic regression analysis	Age, sex, race, hypertension, smoking, diabetes, COPD, CHF, weight loss, ascites, sepsis, dyspnea, renal failure requiring dialysis, use of steroids, ASA classification, functional status, bleeding disorders, type of admission, cancer stage, surgical approach, wound class	Superficial SSI, Right sided Left sided	N=1,270 104 (8.2)	N=2,248 150 (6.7)	N=117 3 (2.6)	N=1,386 31 (2.2)	0.91 (0.89–1.00) 0.36 (0.10–1.35)	0.14 (0.06–0.33) 0.31 (0.18–0.53)	
						Organ/space SSI, Right sided Left sided	73 (5.7)	116 (5.2)	4 (3.4)	43 (3.1)	0.63 (0.07–5.13) 0.63 (0.17–2.26)	0.75 (0.36–1.57) 0.44 (0.26–0.73)	
Koller 2018, USA	2012–2014	Elective colorectal	Retrospective cohort	Propensity score adjusted logistic regression analysis with Bonferroni correction	Not specified	All	N=8,658 1,013 (11.7)	N=11,862 1,210 (10.2)	N=1,232 90 (7.3)	N=10,636 585 (5.5)	0.49 (0.38–0.64)	0.45 (0.40–0.50)	0.91 (0.69-1.20)
Dolejs 2017, USA	2012-2014	Elective colorectal, aged >75 yr	Retrospective cohort	Logistic regression analysis	Adjusted, but not specified for which confounders	Superficial Deep Organ-space^a^	N=1,497 105 (7) 22 (1.5) 60 (4.0)	N=1,788 80 (4.5) 27 (1.5) 68 (3.8)	N=153 4 (2.5) 2 (1.3) 4 (2.5)	N=1,391 28 (2.0) 14 (1.0) 33 (2.4)	0.40 (0.20–1.50) 0.95 (0.15– 3.0) 0.50 (0.20–2.10)	0.41 (0.27–0.62) 0.90 (0.30–2.20) 0.50 (0.40–0.75)	
Garfinkle 2017, USA	2012-2014	Elective colorectal	Retrospective cohort	Logistic regression analysis with Bonferroni correction	Coarsened exact matching on age, ASA classification, chemotherapy, BMI, laparoscopy and ostomy	All	N=13,219 1,903 (14.4)	N=13,935 1,616 (11.6)	N=1,572 137 (8.7)	N=11,720 762 (6.5)	0.62 (0.46–0.87)	0.42 (0.35–0.49)	0.78 (0.55–1.08)
Shwaartz 2016, USA	2012-2014	Elective colorectal, IBD	Retrospective cohort	Logistic regression analysis, multiple outcomes	Adjusted, but not specified for which confounders	Incisional Organ/space	N=1,563 118 (7.5) 126 (8.1)	N=791 59 (7.5) 57 (7.2)	N=325 27 (8.3) 18 (5.5)	N=1,000 48 (4.8) 39 (3.9)	NS	0.55 (0.39–0.79) 0.53 (0.36–0.77)	
Midura 2017, USA	2012-2015	Elective colectomy with anastomosis	Retrospective cohort	Logistic regression analysis	Age, race, diabetes, ASA classification, smoking, disseminated cancer, steroids, renal failure, wound class, chemotherapy, indication surgery, surgical approach, location resection	All	N=11,898 797 (6.7)	N=15,175 895 (5.9)	N=1,791 82 (4.6)	N=16,860 489 (2.9)	0.70 (0.55–0.88)	0.47 (0.42-0.53)	
Kaslow 2018, USA	2012-2015	Elective colorectal	Retrospective cohort	Propensity score adjusted logistic regression analysis.	Age, sex, race, ASA classification, BMI category, >10% weight loss in the last six months, current smoker, hypertension, COPD, dialysis, on steroid for chronic conditions, indication for surgery, approach, operative time	All			N=2,018 171 (8.5)	N=18,576 1117 (6.0)			0.71 (0.60-0.84)
Klinger 2017, USA	2012-2015	Elective colorectal	Retrospective cohort	Propensity score adjusted logistic regression analysis	Age, sex, race, BMI, diabetes, CHF, hypertension, disseminated cancer, steroids, smoking, functional dependence, ASA classification, albumin	Incisional Organ/space	5,471 N/A	7,617 N/A	1,374 N/A	8,855 N/A	0.63 (0.47–0.83) 0.59 (0.41–0.85)	0.39 (0.33–0.46) 0.56 (0.47–0.68)	0.62 (0.46–0.83) 0.93 (0.65–1.35)
Toh 2018, USA	2015	Left-sided colorectal	Retrospective cohort	Logistic regression analysis	Indication, stoma, sex, age, BMI, ASA classification, diabetes, dyspnea, ascites, hypertension, acute renal failure, dialysis, disseminated cancer, prior wound infection, steroids, weight loss, bleeding disorder, transfusion (peri- and postoperative) systemic sepsis, *C. difficile*, albumin, WBC, Ht, operative duration, anastomotic leakage	All	N=1906 N/A	N=1713 N/A	N=199 N/A	N = 2721 N/A	0.50 (0.16–1.54)	0.47 (0.28–0.78)	
Ohman, 2017 USA	2011–2015	Elective colorectal, matched with data from ACS-NSQIP	Single center before after study	Logistic regression analysis	Sex, wound class, ostomy, level of emergency, surgical approach Note: Bowel preparation was part of infection prevention bundle that also included hair removal, skin antisepsis, antibiotic wound irrigation and clean closure	All	N=37 5 (13.5)	N=27 5 (18.5)	N=12 2 (16.7)	N=223 6 (2.7)	1.30 (0.20–7.60)	0.20 (0.10–0.60)	
**Other studies**													
Cannon 2012, Canada	2005–2009	Elective colorectal	Retrospective cohort	Generalized estimated equations	Age, diabetes, COPD wound class, type of resection	All	N=1,978 358 (18.1)	N=3,839 768 (20.0)	N=723 60 (8.3)	N=3,400 311 (9.2)	0.33 (0.21–0.50)	0.43 (0.34–0.55)	
Mulder 2018, The Netherlands	2012–2015	Elective colorectal	Single center before after study	Binomial regression model with a log link function	Age, sex, BMI, perioperative antibiotic prophylaxis, colorectal malignancy, operative time >75^th^ percentile, surgical approach, ASA classification, wound class, surgeon experience	Composite of deep incisional + organ/space SSI and mortality	N=352 50 (14.2)		N=1,058 85 (8.0)		0.58 (0.40–0.79)		

Note. aOR, adjusted odds ratio; ASA, American Society for Anesthesiologists; BMI, body mass index; CDC, Centers for Disease Control and Infection Prevention; CHF, chronic heart failure; CI, confidence interval; Ht, hematocrit; IBD, inflammatory bowel disease; MBP, mechanical bowel preparation; MI, myocardial infarction; N/A, not available; NS, not significant; OAP, oral antibiotic prophylaxis; PE, pulmonary embolism; RCT, randomized controlled trial; SSI, surgical site infection; WBC, white blood cell count.

a (%) SSI and aOR were not reported and estimated from Figure. 1.

## Discussion

In our evaluation of observational studies, OAP reduces the risk of SSI after colorectal surgery by 50%. Combining OAP with MBP had a comparable effect on SSI risk. Although these findings seem conclusive, we must address several limitations. We included only 1 of the studies performed on the ACS-NSQIP database because we were unable to extract the proportion of unique participants across all publications, which inevitably reduced precision. Nevertheless, all studies reported a protective effect of OAP; therefore, we considered the direction of the effect reliable. The magnitude of the effect, however, could not be directly determined because of the limitations that apply to the ACS-NSQIP database, which we believe affected all the studies performed on these data. The database contains a limited number of variables, which likely hampered adequate adjustment for confounders. Thus, residual confounding cannot be excluded. Secondly, the grouping of participants may be unreliable because only MBP administered in the hospital was properly documented. This could imply the presence of misclassification bias when a part of the OAP only group did receive MBP at home. In addition, all studies excluded patients with data missing for the determinant, which may have introduced selection bias. Another issue is that the percentage of patients in the OAP only group was very low compared to the other preparation strategies. Albeit the aORs all demonstrate a protective effect of OAP, several studies clearly lacked power to determine the effectiveness of OAP without MBP. More importantly, these low numbers may also reflect the presence of confounding by indication. The choice of bowel preparation generally depends on surgeon’s preference and on patient’s prognosis. In most studies, a preference for combining OAP with MBP is seen. Not adding MBP to OAP could be because patients are unable to tolerate MBP because they are less fit, or that surgery was performed subacutely. In both cases, SSI risk was higher. This could have led to an underestimation of the effectiveness of OAP only, and it is also impossible to disentangle the impact of MBP when comparing OAP only with OAP and MBP combined because of unknown differences in patient characteristics that influence SSI risk.

That OAP is also effective without MBP was confirmed by a study that investigated OAP in a setting where MBP was not used. In this study, the risk of confounding by indication was present but negligible; OAP was implemented as standard of care.^16^ Although confounding due to residual time variation could not be completely excluded, a reduction in deep SSI and mortality of 42% was reported. Findings from the network meta-analysis also demonstrate that OAP can be administered without MBP. Although this conclusion was based on indirect associations, OAP alone appeared to be a better strategy than OAP with MBP in reducing organ-space infections. In contrast, a single-center RCT from Israel found no difference between OAP and OAP combined with MBP regarding SSI risk, suggesting that the MBP component can be safely omitted.^19^

We also demonstrated that the impact of MBP with OAP is similar to that of OAP alone. Considering the absence of a beneficial effect of MBP alone, the only rationale for continuation of MBP in combination with OAP is because it was hypothesized that the antibiotics were not effective in an uncleansed colon. Based on our findings, we consider the added value of MBP to be questionable at best. This is relevant because, in addition to the higher costs, MBP not only poses a risk of electrolyte disturbance, its administration is also a significant burden to the patient. High-quality evidence is needed to confirm the efficacy of OAP without MBP. An RCT that includes an OAP-only arm and is powered to detect a 40% reduction in SSI risk may bring us closer to closing the research gap on the use of OAP and the necessity of MBP.
